# Resistance to the crayfish plague, *Aphanomyces astaci* (Oomycota) in the endangered freshwater crayfish species, *Austropotamobius pallipes*

**DOI:** 10.1371/journal.pone.0181226

**Published:** 2017-07-27

**Authors:** Laura Martín-Torrijos, Miquel Campos Llach, Quim Pou-Rovira, Javier Diéguez-Uribeondo

**Affiliations:** 1 Department of Mycology, Real Jardín Botánico (RJB-CSIC), Plaza Murillo, 2, Madrid, Spain; 2 Consorci del´Estany, Plaça dels estudis, 2, Banyoles, Girona, Spain; Uppsala University, SWEDEN

## Abstract

The pathogen *Aphanomyces astaci* Schikora 1906 is responsible for the decline of the native crayfish species of Europe, and their current endangered status. This pathogenic species is native to North America and only colonizes aquatic decapods. The North American crayfish species have a high resistance to this pathogen, while species from other regions are highly susceptible. However, recent field and laboratory observations indicate that there might exist some populations with resistance against this disease. The objective of this study was to test the susceptibility of 8 selected native European crayfish populations of *Austropotamobius pallipes* Lereboullet 1858 from the Pyrenees. We challenged them against the genome sequenced strain AP03 of *A*. *astaci* isolated from a North American red swamp crayfish, *Procambarus clarkii* Girard 1852, in the Garrotxa Natural Park, Girona. The results showed that there are significant differences (P<0,001) among populations, although most of them show high mortality rates after the zoospore challenge with *A*. *astaci*. However, one population from Girona exhibited a 100% survival during a four-month monitoring period under the experimental conditions tested. Histological analyses revealed a high immune reaction in tissues examined, *i*.*e*., encapsulation and melanization of hyphae, similar to that found in North American resistant crayfish species. These results represent the first observation of a native European crayfish population showing high resistance towards the most virulent genotype of this pathogen, *i*.*e*., genotype Pc. The identification of this population is of key importance for the management of these endangered species, and represents a crucial step forward towards the elucidation of the factors involved in the immune reaction against this devastating pathogen.

## Introduction

Fungal and fungal-like emerging infectious diseases (EIDs) are seriously affecting endangered wildlife species and present a major concern for biodiversity conservation. As a result, EIDs are currently attracting an important number of scientific efforts and attention [1–3]. So far, one of the most devastating EIDs is the crayfish plague caused by the organism *Aphanomyces astaci* Schikora 1906 (Oomycota). This pathogen is listed among the 100 World´s Worst Invasive Alien Species [4] and is responsible for the destruction of European freshwater crayfish populations since its introduction in Europe in the late 19th century [5].

The crayfish plague constitutes a classic example of a disease emergence as consequence of introducing alien species into a new biogeographic region [6]. This pathogen originates from North America, where it coexists with their natural hosts, *i*.*e*., the freshwater crayfish [1]. In North American crayfish, their interaction with *A*. *astaci* seems to be a balanced relationship as a result of a shared history of coevolution [7]. Thus, the pathogen is usually unable to entirely colonize its host because their immune system can encase the pathogen within the cuticle by increasing its phenoloxidase activity [8]. This enzyme produces melanin as an end product, which has both fungitoxic and fungistatic activity [8]. The equilibrium prevents the pathogen from overtaking its host but instead the host becomes chronically infected. However, in native European crayfish species, the immune system does not respond as efficiently to prevent the spread of *A*. *astaci* and are, therefore, highly vulnerable to the disease [9]. Thus, in susceptible species, this pathogen can kill up to 100% of the individuals infected [7]. The hyphae of the pathogen can colonize the host almost without opposition and results in death within a few days [10]. In this case, no melanization, *i*. *e*., immune system response, is usually seen, and there are only few reports of partial melanization of the colonizing hyphae. Most of these cases have been reported in the native European species, *Astacus astacus*, Linnaeus 1758 originating from Northern Europe, and these cases seem to be caused by low virulent strains of *A*. *astaci* [11–13].

So far, 5 genotypes of *Aphanomyces astaci* have been characterized, *i*.*e*., As, PsI, PsII, Pc and Or [14–16]. The original hosts of these genotypes are North American crayfish species; however, the original host of the genotype As has not identified found yet [14–16]. The original host of strains corresponding to genotypes PsI and PsII is the crayfish species *Pacifastacus leniusculus* Dana 1852 [14], while the original hosts of the genotypes Pc and Or are *Procambarus clarkii* Girard 1852 and *Orconectes limosus* Rafinesque 1817, respectively [15, 16]. Isolates corresponding to the genotype As are thought to be responsible for the first outbreaks of the crayfish plague in Europe during the 19th century [14] as a consequence of deliberated introduction of freshwater crayfish from North America into Europe. Recent studies indicate that some isolates of this genotype possess low virulence [11].

The impact of crayfish plague in Europe has been devastating and has decreased not only crayfish populations and their fisheries but also altered the ecology of freshwater ecosystems[17, 18]. In Spain, since the introduction in 1973 of *P*. *leniusculus* and *P*. *clarkii*, the native crayfish, *Austropotamobius pallipes* Lereboullet 1858, have drastically declined [18]. Currently, this species is listed as at “risk of extinction” or “endangered” because of the limited number of remaining populations, and the constant threat and spread of crayfish plague carriers [19–21]. There are no efficient treatments to overcome this disease, and the only efficient measures to control it are, so far, those that aim to prevent the spread of their carriers. A better understanding of the mechanism of disease resistance represents one of the first steps to designing new strategies to prevent disease development. Increased activation of the innate and adaptive immune defenses by pathogen exposure has recently been described for diverse species, *e*.*g*., amphibians or rodents. Thus, some amphibians native to Australia, Panama and South Africa have developed an increased resistance to the fungus *Batrachochytrium dendrobatidis* Longcore, Pessier & D.K. Nichols 1999 [22, 23], and some Australian rabbits have increased their resistance towards hemorrhagic disease virus (RHDV) by implementing of a whole complex of inter-related defenses [24].

Recent studies on native European crayfish appear to show that some Finnish wild populations might have an increased resistance towards the less virulent genotype of *A*. *astaci* [11, 25]. During the last decade we have observed a number of crayfish plague events involving native Pyrenean populations of *A*. *pallipes*. Interestingly, Pc infected specimens from these populations show melanization and have longer survival [21] (Jokin Larumbe and Joan Montserrat, personal communication). However, there are no comparative studies regarding pathogen resistance in European crayfish populations towards the *A*. *astaci* Pc genotype. Lack of dedicated studies precipitated our investigation into a number of potential resistant European populations against *A*. *astaci*. Therefore, the objective of this study is to test the susceptibility and resistance of geographically isolated native populations of *A*. *pallipes* from diverse Pyrenean areas, to the Pc highly virulent strain of *A*. *astaci*.

## Material and methods

### Ethical statement

All experimental procedures and animal manipulations, as well as field sampling, were performed according to the EU and Spanish legislation. All experiments were approved and carried out according to the regulations of Spanish Ministry MINECO and Local Authorities of the Gobierno de Navarra and Generalitat de Catalunya. Furthermore, the crayfish specimens used in this study were provided by Gobierno de Navarra (2 populations from the Western Pyrenees) and by Generalitat de Catalunya (6 populations from the Eastern Pyrenees) which are the Authorities entitled to collect samples of endangered species, *i*. *e*., *Austropotamobius pallipes*, in Spanish watercourses. No additional permits were required for the described field or laboratory studies, since the ethics approval in the Spanish law is not required for working with arthropod invertebrates. Moreover, this study was carried out in strict accordance with the recommendations and the protocols stablished in previous studies to minimize suffering, *i*. *e*., euthanised by exposure to chloroform vapours [26].

### Crayfish sampling

**Fig 1:**
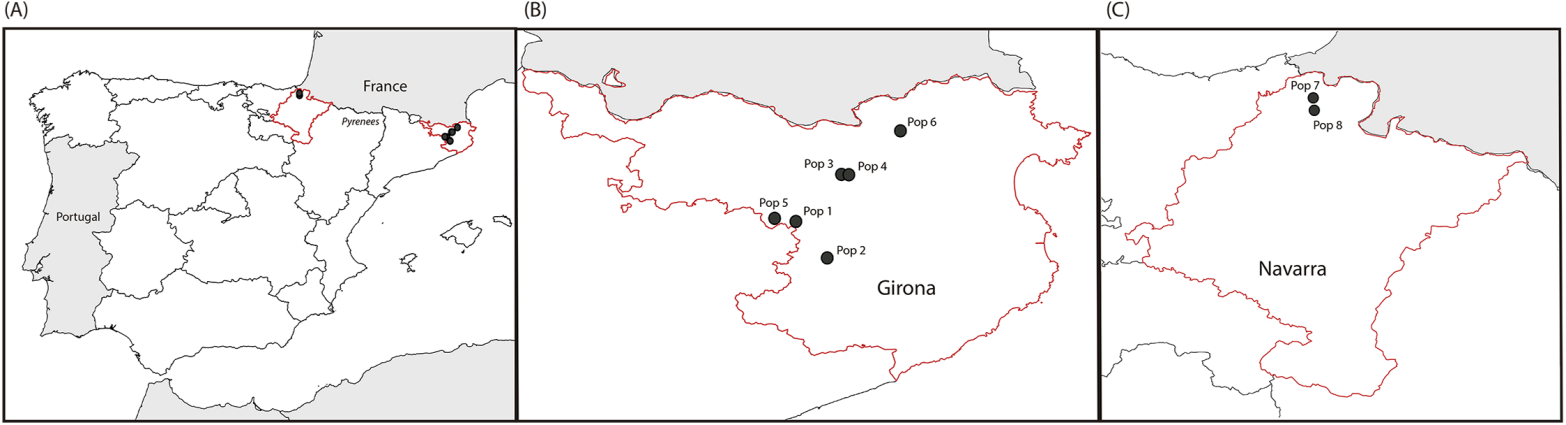
Location of the selected *Austropotamobius pallipes* populations across the Pyrenees. (A) Distribution of the eight selected populations of *Austropotamobius pallipes* from the Spanish Pyrenees. These originate from the: (B) Eastern Pyrenees, Girona (Pop 1: La Fábrega, Pop 2: Escaramart, Pop 3: La Plana, Pop 4: Santa Llúcia, Pop 5: Falgars and Pop 6: La Muga), and (C) Western Pyrenees, Navarra (Pop 7: Doneztebe and Pop 8: Sunbilla).

Specimens of the native European crayfish, *A*. *pallipes*, were collected from eight selected native populations in the Pyrenees mountain range (Spain) (Fig 1A). None of the selected populations included in this study cohabit with any North American crayfish species. Some of these populations, *i*.*e*., Doneztebe, La Muga, were suspected to have higher resistance to *A*. *astaci* based on reports from staff of the Biodiversity Service, Government of Navarra and Natural Park of La Garrotxa, Girona (Jokin Larumbe and Joan Montserrat, *personal communication*). Populations were obtained from Eastern (six) and Western (two) Pyrenees (Fig 1B and 1C and Table 1) and only males were used in the experiments. Each population was marked by clipping with scissors the lateral tips of selected dorsal abdomen segments. Specimens from each population were first kept in separated 0.80m x 0.50m x 0.40 m aquaria each containing 50 liters of aerated filtered pond water and hiding places. The specimens were maintained for two weeks to ensure the acclimatization and were fed weekly with potato and monitored daily to remove dead crayfish or excess dirtiness.

**Table 1:** Location and number of the selected *Austropotamobius pallipes* populations of the Pyrenees.

	Location	Replicate 1	Replicate 2	Replicate 3	Control
**Pop 1**	La Fábrega	5	5	5	5
**Pop 2**	Escaramart	4	4	5	5
**Pop 3**	La Plana	5	5	5	5
**Pop 4**	Santa Llúcia	5	5	5	5
**Pop 5**	Falgars	5	5	5	5
**Pop 6**	La Muga	5	5	5	5
**Pop 7**	Doneztebe	5	5	5	5
**Pop 8**	Sunbilla	3	5	1	5
**Total**		**37**	**39**	**36**	**40**

### Zoospore production and *Aphanomyces astaci*-challenge experiments

Five individuals from each population were transferred and pooled into four experimental aquaria (40 specimens each, five specimens from each population) maintained under the same conditions as described above. In order to ensure a suitable temperature, oxygen levels and water quality, the aquaria were checked daily before and during the experiment. Water was replaced weekly to remove excess dirtiness and to avoid cannibalism, dead individuals were removed once they were found. During the acclimatization period some individuals died. For this reason, the experiments had different number of individuals (Table 1).

For the challenge experiments, the AP03 strain of *A*. *astaci*, recently genome sequenced (GenBank accession number KX405004), was used [27]. This strain was isolated from specimens of a population of *P*. *clarkii* located in the surroundings of the Garrotxa Natural Park [27]. This strain belongs to the Pc genotype and is deposited at the RJB-CSIC culture collection. Zoospores were produced by following the protocol described by Diéguez-Uribeondo et *al*. 1995 [15] and added gently into three of the four aquaria, maintained as above (replicates 1, 2 and 3, hereafter), until reaching a concentration of 10^2^ zoospores mL^-1^. The number of crayfish from each population is listed in Table 1. A control was also run in a separated aquarium that had 40 crayfish (five specimens from each population). No zoospores were added into this aquarium (Table 1). The experiment was followed for 120 days and the aquaria were examined on a daily basis.

For further microscopic and molecular analyses, surviving crayfish from replicates 1, 2 and 3, as well as the crayfish from control aquarium were euthanized by exposure to chloroform vapors [26] after 120 days from the challenge experiments.

### Macroscopic and microscopic examination

The crayfish did not show any melanization before the experiments. All crayfish were checked upon arrival to the laboratory and before being challenged with zoospores. Crayfish were checked daily for disease symptoms and dead crayfish were removed and examined for the presence of melanized areas both macroscopically and microscopically. For microscopic examination, the subabdominal cuticle was removed and observed using an inverted microscope Olympus CKX41SF (Olympus Optical, Tokyo, Japan). Light micrographs were captured using a Qimaging Micropublisher 5.0 digital camera (Qimaging, Burnaby, BC, Canada). Digital image analysis was performed using the software Syncroscopy-Automontage (Microbiology International Inc., Frederick, MD) as described in Diéguez-Uribeondo et *al*. 2003 [28]. After 120 days of the challenge experiments, live crayfish were euthanized [26] and examined as described above.

### Molecular analyses

All the crayfish were tested for the presence of *A*. *astaci* before and after the challenge experiment by applying molecular PCR-based tests [26] to selected tissues: a walking leg before the challenge and subabdominal cuticle, telson, swimmerets, and eye after it, in order to certify that all the crayfish used in the challenge experiment were crayfish plague free. Genomic DNA was extracted by using an E.Z.N.A.® Insect DNA Kit (Omega bio-tek, Norcross, Atlanta, USA). DNA extractions and *A*. *astaci* diagnostic primers 42 [26] and 640 [29] (including the ITS1 and ITS2 sourrounding the 5.8S rDNA, and anchored in ITS1 and ITS2 regions, respectively) were used for a single round PCR according to the assay described by Oidtmann et *al*. 2006 [26]. Positive and negative controls (containing DNA extracted from a AP03 pure culture and containing no DNA, respectively) were included. Three-μl aliquots of the amplification product were analyzed by running an electrophoresis in 1% agarose TAE gels stained with SBYR® Safe (Thermo Fisher Scientific). Double strand PCR positive products were sequenced using an automated sequencer (Applied Biosystems 3730xl DNA, Macrogen, Netherlands). Each sequence strand was assembled and edited using the program Geneious v6.14 [30]. We ran a BLAST search to check the nature of the generated sequences in order to verify the coincidence with the strain used in the zoospore challenge, *i*.*e*., AP03 isolate.

### Assessment of cumulative mortality rates

Cumulative mortality rates were followed over a period 120 days after challenging the crayfish with *A*. *astaci* zoospore. Cumulative mortality rates were calculated as the number of dead crayfish per total crayfish challenged. In order to minimize secondary infections from diseased dead crayfish, these were removed from the aquarium immediately after death and the water was changed every week.

### Statistical analyses

A Kaplan-Meier Log-Rank (Mantel-Cox) was performed in R Development Core Team to evaluate differences in cumulative mortality rates among populations. A General Linear Model Test (GLM) was performed in SAS to compare population means across replicates, as well as a Least Significance Difference (LSD) t-test to determine differences between populations at selected time periods.

## Results

### Macroscopic and microscopic examinations and assessment of cumulative mortality rates

**Fig 2:**
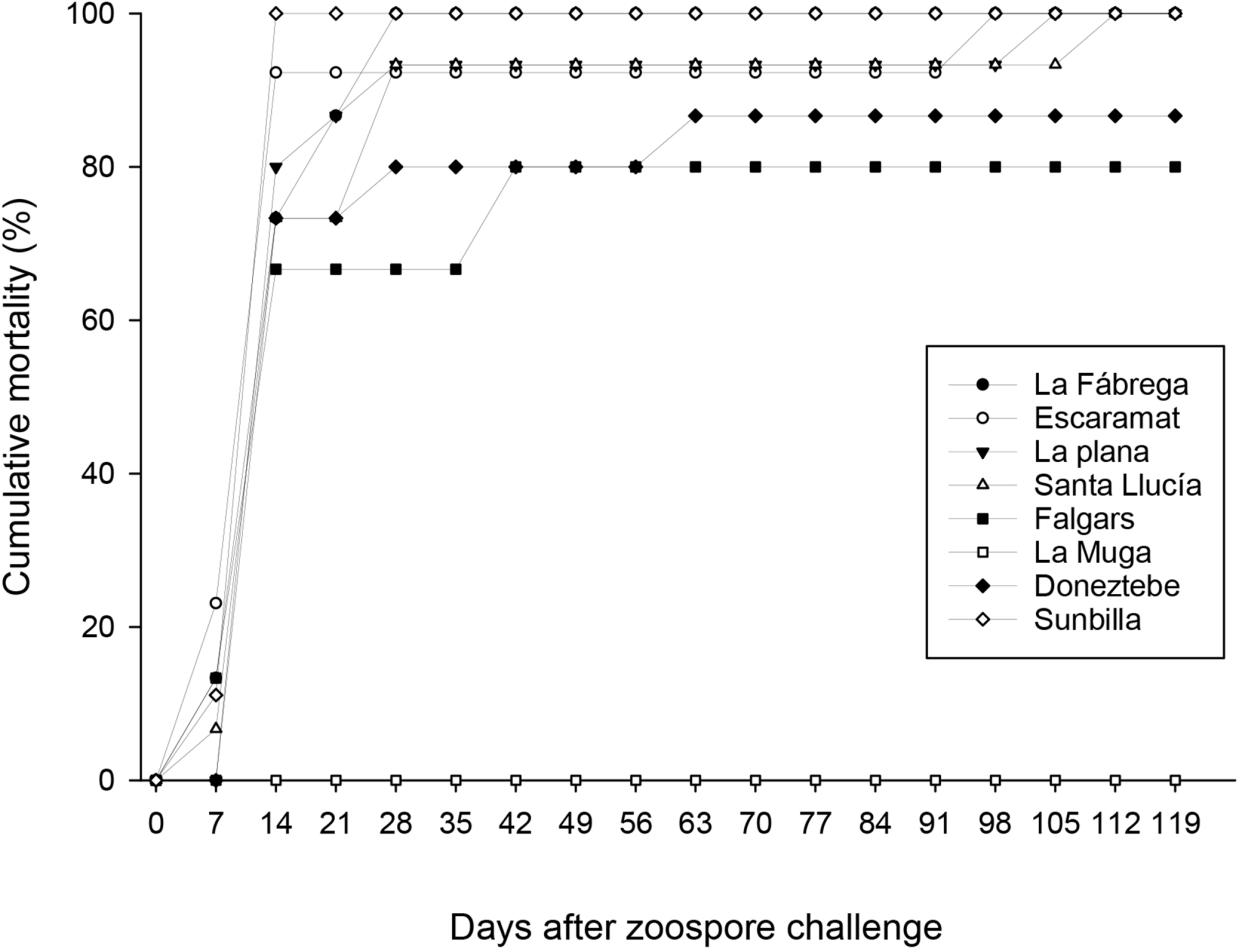
Cumulative mortality in *Austropotamobius pallipes* populations using *Aphanomyces astaci* zoospores. Challenge experiments were carried out in eight selected populations of *Austropotamobius pallipes* (Pop 1: La Fábrega, Pop 2: Escaramart, Pop 3: La Plana, Pop 4: Santa Llúcia, Pop 5: Falgars, Pop 6: La Muga, Pop 7: Doneztebe and Pop 8: Sunbilla) during 120 days after the zoospore challenge. No crayfish from the control died during the experimental period of 120 days. The control is not represented in the figure.

Crayfish specimens tested from all populations challenged with zoospore of *A*. *astaci* start dying on day 7 and the period of crayfish mortality lasted 102 days after the initiation of the experiment. However, all specimens from the population of La Muga (Pop 6) and the control survived for the whole experimental period of 120 days. One main mortality event occurred between day 7 and 14 after zoospore challenge. In this period, a total of 66 crayfishes died (16 crayfish from replicate 1, 30 crayfishes from replicate 2, and 30 crayfishes from replicate 3). Subsequent isolated deaths were observed till the end of the experiment (8 from replicate 1 and one individual from replicate 2) (Fig 2). At day 120 after zoospore challenge, three crayfishes from the population of Falgars, two from population of Doneztebe, and all from population of La Muga and the control survived.

**Fig 3:**
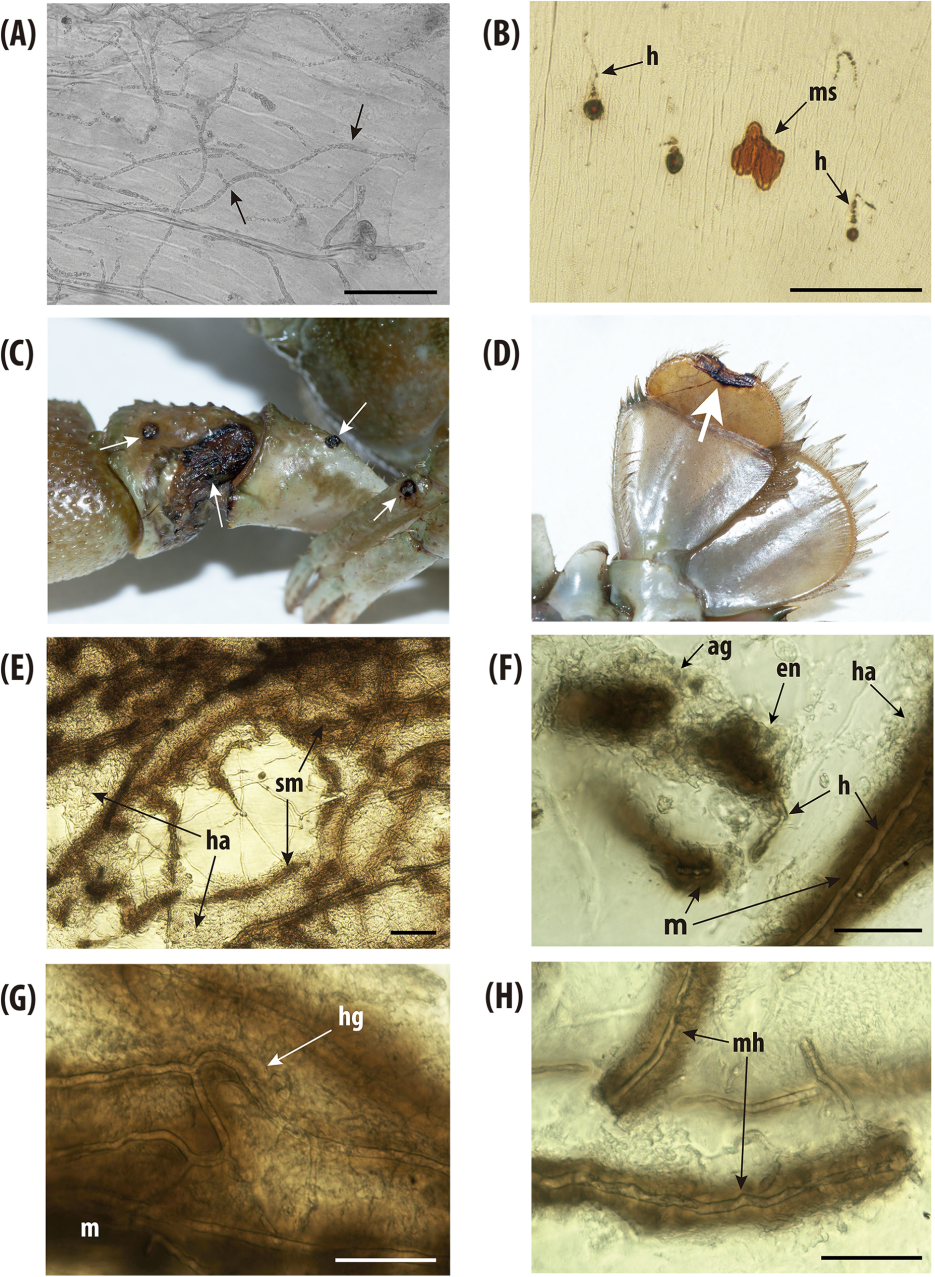
*Aphanomyces astaci* colonization and immune reaction in susceptible and resistant crayfish *Austropotamobius pallipes*. Micrographs of susceptible crayfish dying after being challenged with *A*. *astaci* zoospores: (A) soft abdominal cuticle showing the growth of non-melanized hyphae (arrow); and (B) melanized spots (ms) and hyphae (h) occasionally seen in susceptible crayfish. Macroscopic signs of infection in crayfish with increased-resistance against *A*. *astaci*: (C) strong immune reaction, *i*.*e*., melanin formation, against infection by *A*. *astaci* visualized as large melanized patch (arrows) on the joint of a chela, and also on a walking leg; and (D) a melanized patch (arrow) in the telson. Micrographs showing the immune response process against *A*. *astaci* in crayfish with increased-resistance: (E) general view of an infected cuticle showing aggregation of haemocytes (ha) and strong melanization (sm) of an infected area; (F) haemocytes (ha) aggregating (ag) and encapsulating (en) *A*. *astaci* hyphae (h) that eventually produce melanin (m) deposits; (G) general view of an infected area showing hyphal growth (hg) inhibition by producing heavy melanin (m) deposits; and (H) detailed view of heavily melanized hyphae (mh). Bar = 100 μm.

Macroscopic examination of dead crayfish from all periods and all populations did not reveal the presence of melanized spots on the cuticle. Microscopic observations showed the presence of abundant non-melanized hyphae with round tips and homogenous diameter, ca 10 um, characteristic of *A*. *astaci* infection, growing within the subabdominal cuticle (Fig 3A). The presence of melanized hyphae and micro-melanized spots of a ca 30 μm were only seen occasionally (Fig 3B). Macroscopic examinations of specimens from La Muga (Pop 6) showed the presence of melanized patches on the joints of the walking legs, carapace, uropods and soft ventral cuticle (Fig 3C and 3D). Microscopically, the subabdominal cuticle of these specimens possessed strongly melanized hyphae characteristic of an *A*. *astaci* chronic infection (Fig 3E). Different stages of hyphal encapsulation by haemocytes (Fig 3F) were seen, ranging from weak melanized to completely melanin-covered hyphae (Fig 3G and 3H).

Surviving crayfish from Falgars (Pop5) and Doneztebe populations (Pop7) did not show macroscopic signs of melanization. At microscopic level, melanized spots were often seen, and when hyphae were observed, they always appeared partially melanized.

### Survivable statistical contrasts

The survival analysis (Kaplan-Meier Log-Rank (Mantel-Cox) established statistically significant differences between the cumulative rates of the 8 populations studied (Pop 1-Pop 8) (χ_2_ = 62.4 on 7 degrees of freedom, *p* = 4.91e-11) during the 120 days experiment. The GLM model determined statistically significant differences (*F* = 9.42, *p*<0.0001). A closer look showed statically significant differences between populations (*F* = 26.70; *p*<0.0001) and replicates (*F* = 4.96, *p*<0.009), but the non-interaction between replicates and populations (*F* = 1.41; *p*>0.05) allowed us to combine the data from the three replicates. LSD t-test, for comparing population means, showed significant differences between populations (*p*<0.05), allowing the establishment of different groups according to the survivorship (Table 2).

**Table 2:** LSD t-test on survivorship rates of 8 selected populations of *Austropotamobius pallipes* challenged with *Aphanomyces astaci* zoospores. Populations are grouped (A; B; BC: BCD; CD; D) by their survivorship rates (means with the same letter are not significantly different).

	*n*	Mean	t-Grouping		
**Pop 6**	15	90.000	A		
**Pop 5**	15	28.533	B		
**Pop 7**	15	23.600	B	C	
**Pop 4**	15	16.400	B	C	D
**Pop 3**	15	15.733	B	C	D
**Pop 2**	13	14.077	B	C	D
**Pop 1**	15	11.533		C	D
**Pop 8**	9	8.154			D

### Molecular analyses

All the crayfish tested negative for the presence of *A*. *astaci* when using specific PCR Test before the challenge with *A*. *astaci* zoospores. Tissues from dead and sacrificed crayfish of the 3 replicates exposed to *A*. *astaci* zoospores challenge tested positive for *A*. *astaci* when using the specific PCR Test. Tissues of the control crayfish always tested negative. Alignment of 112 obtained DNA sequences from positive crayfish revealed that all were identical to the isolate of *A*. *astaci* used for the zoospore challenge experiment and a BLAST search of the sequence of isolates showed 100% similarity to AP03 isolate.

## Discussion

This study identifies for the first time a population of native European freshwater species showing a 100% survival against the infection by the crayfish plague pathogen *A*. *astaci*. This *A*. *astaci*-resistant population was first suspected in nature and now confirmed under laboratory conditions with the most virulent strain known [15]. After *A*. *astaci* zoospore challenge, all crayfish from population of La Muga tested positive for the pathogen. The immune response observed in the crayfish cuticle, *i*. *e*., encapsulation and strong melanization of hyphae, is similar to that exhibited by North American freshwater crayfish [31]. This shows that crayfish from La Muga became infected by the pathogen and its colonization was eventually contained.

The high resistance in North American crayfish seems to be the consequence of a constantly activated pro-Phenoloxidase system (pro-Po system) [10]. This immune response enables them to react rapidly against pathogens [10, 32]. In European crayfish populations, however, the activation of the pro-Po system is not efficient enough against this pathogen and crayfish eventually die from this disease [10]. The key mechanisms that enable the crayfish from La Muga to become resistant are still unknown and need to be further investigated. The enzymes related to the formation of melanin deposits are potential candidates to be investigated [33]. Thus, enzymes from the cascade of serine proteinases that control the cleavage of the pro-form of the pro-Po system-activating enzyme (pro-ppA) into active ppA, should be tested in the near future [33]. Moreover, comparative studies on functional genomics can now be performed and facilitate the elucidation of the mechanisms and factors favoring the defense against this severe pathogen.

We also found differences in cumulative mortality at individual level within two populations, *i*.*e*., Falgars and Doneztebe. Some specimens could survive longer periods than others, and even resist the whole period of the experiment, *i*.*e*., 120 days. Moreover, these crayfish also tested positive to *A*. *astaci*, and showed an evident immune response *i*.*e*., melanized spots and partially melanized hyphae. This suggests that the mechanism for resistance might also be present not only in a particular population but also in some specimens from geographically isolated populations of *A*. *pallipes*.

Longer survival periods and an increased melanization response against *A*. *astaci* have been reported in populations of the also native European species *A*. *astacus* in Finland [11, 13, 25, 34]. Some crayfish survived for longer periods after the exposure to zoospore concentrations of the low virulent strains of *A*. *astaci*, *i*.*e*., genotype As [11, 25]. Moreover, recent studies on other native European crayfish species, *e*.*g*., *A*. *astacus*, *Astacus leptodactylus* Eschscholtz 1823 and *Austropotamobius torrentium* Schrank 1803 from Finland, Turkey, and Slovenia, respectively, detected some specimens that chronically carry the low virulent genotype As [11, 12, 35, 36]. This genotype seems to have been introduced into Europe during the 19th century [9, 14]. Some authors have speculated that a decrease virulence/increased immune response is the consequence of a rapid co-evolution of the introduced *A*. *astaci* and its new European host [36]. Because *A*. *astaci* is highly specialized on freshwater crayfish and highly virulent on susceptible species [9], low-virulent strains appear to have avoided evolutionary suicide by coexisting for a longer period with their hosts, in comparison with the high-virulent strains that rapidly kill their hosts [12]. This balance has already been observed in other host-pathogen interactions. For example, some Australian and Panamanian amphibian species have recently showed resistance to chytridiomycosis as a result of specific skin peptides that inhibit its growth [23].

Our investigation, however, presents a different finding since it identifies a crayfish population with increased resistance to a highly virulent strain, *i*. *e*., genotype Pc [15] in a species so far characterized as highly susceptible [9] and in a geographical location where the pathogen has been recently introduced, *i*.*e*., 1990´s [21]. At least two hypotheses can be raised to explain the rapid development of *A*. *astaci*-resistance in this population: (i) this might be an inherent genetic property of this population, since it belongs to a different lineage than the other populations tested [37]; and (ii) continuous crayfish plague events favored by the high density of *P*. *clarkii* in La Muga area [21] might have acted as a selective pressure promoting this resistant phenotype. Additional comparative analyses using other populations of la Muga *A*. *pallipes* lineage might confirm one or the other hypothesis.

The finding of a population of *A*. *pallipes* with high resistance to *A*. *astaci* can be very helpful for the conservation management of these endangered species in Europe, since: (i) it opens the window for the existence of other resistant populations and specimens so far overlooked, and sets the basis for the identifications of new resilient lineages; (ii) it allows designing breeding programs oriented to raise *A*. *astaci-*resistant lineages as a preventive measure against the extinction of this species, and (iii) it is a crucial step forward to the elucidation of the key factors involved in the defense reaction against this devastating pathogen as mentioned above. In a broader context, this discovery is also significant considering the current scenario of globalization that allows invasive species, such as *A*. *astaci* chronic carriers, to reach the areas of distribution of susceptible species, *i*.*e*., East Asia, South America, Madagascar, or Oceania, some of them identified as “hot spots” of crayfish biodiversity. Currently, breeding and conservation programs are being implemented by Generalitat de Catalunya to preserve this *A*. *pallipes* population, and to carry out further investigations in order to understand the resistant mechanisms and genetics involved in this resistant population.

## References

[1] Daszak P, Cunningham AA, Hyatt AD. Emerging Infectious Diseases of Wildlife—Threats to Biodiversity and Human Health. Sci. 2000;287(5452):443–9. doi: 10.1126/science.287.5452.443 10.1126/science.287.5452.443 10642539

[2] Jones KE, Patel NG, Levy MA, Storeygard A, Balk D, Gittleman JL, et al Global trends in emerging infectious diseases. Nat. 2008;451(7181):990–3. http://www.nature.com/nature/journal/v451/n7181/suppinfo/nature06536_S1.html. 10.1038/nature06536 PMC5960580 18288193

[3] Racaniello VR. Emerging infectious diseases. Journal of Clinical Investigation. 2004;113(6):796–8. doi: 10.1172/JCI21370 15067308 10.1172/JCI21370 PMC362132

[4] Lowe S, Browne M, Boudjelas S, De Poorter M. 100 of the World’s Worst Invasive Alien Species A selection from the Global Invasive Species Database.: The Invasive Species Specialist Group (ISSG) a specialist group of the Species Survival Commission (SSC) of the World Conservation Union (IUCN). 2000. p. 12.

[5] Unestam T. Defence reactions in and susceptibility of Australian and New Guinean freshwater crayfish to European-crayfish-plague fungus. Aust J Exp Biol Med. 1975;53(5):349–59. 10.1038/icb.1975.40 820321

[6] Alderman DJ. Geographical spread of bacterial and fungal diseases of crustaceans. Scientific and Technical Review of the Office International des Epizooties. 1996;15(2):603–32. 10.20506/rst.15.2.943 8890383

[7] Unestam T. Resistance to the crayfish plague in some American, Japanese and European crayfishes. Drottningholm: 1969.

[8] Söderhäll K, Häll L, Unestam T, Nyhlén L. Attachment of phenoloxidase to fungal cell walls in arthropod immunity. Journal of Invertebrate Pathology. 1979;34(3):285–94. http://dx.doi.org/10.1016/0022-2011(79)90075-2.

[9] Rezinciuc S, Sandoval-Sierra JV, Oidtmann B, Diéguez-Uribeondo J. The Biology of Crayfish Plague Pathogen Aphanomyces astaci. Current Answers to Most Frequent Questions In: Kawai T, Faulkes Z, Scholtz G, editors. Freshwater Crayfish: A Global Overview: CRC Press; 2015 p. 182–204.

[10] Cerenius L, Bangyeekhun E Fau—Keyser P, Keyser P Fau—Soderhall I, Soderhall I Fau—Soderhall K, Soderhall K. Host prophenoloxidase expression in freshwater crayfish is linked to increased resistance to the crayfish plague fungus, Aphanomyces astaci. 2003;(1462–5814 (Print)). 10.1046/j.1462-5822.2003.00282.x 12713493

[11] Makkonen J, Jussila J, Kortet R, Vainikka A, Kokko H. Differing virulence of Aphanomyces astaci isolates and elevated resistance of noble crayfish Astacus astacus against crayfish plague. Dis Aquat Organ. 2012;102(2):129–36. doi: 10.3354/dao02547 23269387 10.3354/dao02547

[12] Jussila J, Makkonen J, Vainikka A, Kortet R, Kokko H. Latent crayfish plague (Aphanomyces astaci) infection in a robust wild noble crayfish (Astacus astacus) population. Aquaculture. 2011;321(1–2):17–20. http://dx.doi.org/10.1016/j.aquaculture.2011.08.026.

[13] Viljamaa-Dirks S, Heinikainen S, Nieminen M, Vennerström P, Pelkonen S. Persistent infection by crayfish plague Aphanomyces astaci in a noble crayfish population–a case report. Bulletin of the European Association of Fish Pathologists. 2011;31(5):182–8.

[14] Huang T-s, Cerenius L, Söderhäll K. Analysis of genetic diversity in the crayfish plague fungus, Aphanomyces astaci, by random amplification of polymorphic DNA. Aquaculture. 1994;126(1):1–9. http://dx.doi.org/10.1016/0044-8486(94)90243-7.

[15] Diéguez-Uribeondo J, Huang T-S, Cerenius L, Söderhäll K. Physiological adaptation of an Aphanomyces astaci strain isolated from the freshwater crayfish Procambarus clarkii. Mycological Research. 1995;99(5):574–8. http://dx.doi.org/10.1016/S0953-7562(09)80716-8.

[16] Kozubikova E, Viljamaa-Dirks S, Heinikainen S, Petrusek A. Spiny-cheek crayfish Orconectes limosus carry a novel genotype of the crayfish plague pathogen Aphanomyces astaci. J Invertebr Pathol. 2011;108(3):214–6. doi: 10.1016/j.jip.2011.08.002 . 21856310 10.1016/j.jip.2011.08.002

[17] Füreder L, Gherardi F, Holdich D, Reynolds J, Sibley P, Souty-Grosset C. Austropotamobius pallipes. The IUCN Red List of Threatened Species 2010: e.T2430A9438817 2010 [cited 2016 28 February 2016.]. Available from: http://www.iucnredlist.org/details/2430/0.

[18] Alonso F, Temiño C, Diéguez-Uribeondo J. Status of the white-clawed crayfish, Austropotamobius pallipes (Lereboullet, 1858), in Spain: distribution and legislation. Bulletin Français de la Pêche et de la Pisciculture 2000;356 31–53.

[19] Diéguez-Uribeondo J, Temiño C, Múzquiz JL. The crayfish plague fungus (Aphanomyces astaci) in Spain. Bull Fr Pêche Piscic. 1997;(347):753–63.

[20] Dieguez-Uribeondo J. Dispersion of the Aphanomyces astaci-carrier Pacifastacus leniusculus by humans represents the main cause of disappearance of the indigenous crayfish of Navarra. Bull Fr Pêche Piscic. 2005:380–1.

[21] Rezinciuc S, Galindo J, Montserrat J, Dieguez-Uribeondo J. AFLP-PCR and RAPD-PCR evidences of the transmission of the pathogen Aphanomyces astaci (Oomycetes) to wild populations of European crayfish from the invasive crayfish species, Procambarus clarkii. Fungal Biol. 2014;118(7):612–20. doi: 10.1016/j.funbio.2013.10.007 . 25088075 10.1016/j.funbio.2013.10.007

[22] Ramsey JP, Reinert LK, Harper LK, Woodhams DC, Rollins-Smith LA. Immune Defenses against Batrachochytrium dendrobatidis, a Fungus Linked to Global Amphibian Declines, in the South African Clawed Frog, Xenopus laevis. Infection and Immunity. 2010;78(9):3981–92. doi: 10.1128/IAI.00402-10 20584973 10.1128/IAI.00402-10 PMC2937463

[23] Woodhams DC, Ardipradja K, Alford RA, Marantelli G, Reinert LK, Rollins-Smith LA. Resistance to chytridiomycosis varies among amphibian species and is correlated with skin peptide defenses. Anim Conserv. 2007;10(4):409–17. doi: 10.1111/j.1469-1795.2007.00130.x

[24] Elsworth PG, Kovaliski J, Cooke BD. Rabbit haemorrhagic disease: are Australian rabbits (Oryctolagus cuniculus) evolving resistance to infection with Czech CAPM 351 RHDV? Epidemiol Infect. 2012;140(11):1972–81. doi: 10.1017/S0950268811002743 . 22244198 10.1017/S0950268811002743

[25] Makkonen J, Kokko H, Vainikka A, Kortet R, Jussila J. Dose-dependent mortality of the noble crayfish (Astacus astacus) to different strains of the crayfish plague (Aphanomyces astaci). J Invertebr Pathol. 2014;115:86–91. doi: 10.1016/j.jip.2013.10.009 . 24184185 10.1016/j.jip.2013.10.009

[26] Oidtmann B, Geiger S, Steinbauer P, Culas A, Hoffmann RW. Detection of Aphanomyces astaci in North American crayfish by polymerase chain reaction. Dis Aquat Organ. 2006;72:53–64. doi: 10.3354/dao072053 17067073 10.3354/dao072053

[27] Makkonen J, Vesterbacka A, Martin F, Jussila J, Dieguez-Uribeondo J, Kortet R, et al Mitochondrial genomes and comparative genomics of Aphanomyces astaci and Aphanomyces invadans. Sci Rep. 2016;6:36089 doi: 10.1038/srep36089 ; PubMed Central PMCID: PMCPMC5093560. 27808238 10.1038/srep36089 PMC5093560

[28] Diéguez-Uribeondo J, Förster H, Adaskaveg JE. Digital Image Analysis of Internal Light Spots of Appressoria of Colletotrichum acutatum. Biochemistry and Cell Biology. 2003;93(8):923–30. 10.1094/PHYTO.2003.93.8.923 18943858

[29] Oidtmann B, Schaefers N, Cerenius L, Soderhall K, Hoffmann RW. Detection of genomic DNA of the crayfish plague fungus Aphanomyces astaci (Oomycete) in clinical samples by PCR. Vet Microbiol. 2004;100(3–4):269–82. doi: 10.1016/j.vetmic.2004.01.019 . 15145505 10.1016/j.vetmic.2004.01.019

[30] Kearse M, Moir R, Wilson A, Stones-Havas S, Cheung M, Sturrock S, et al Geneious Basic: an integrated and extendable desktop software platform for the organization and analysis of sequence data. Bioinformatics. 2012;28(12):1647–9. doi: 10.1093/bioinformatics/bts199 ; PubMed Central PMCID: PMCPMC3371832. 22543367 10.1093/bioinformatics/bts199 PMC3371832

[31] Nyhlén L, Unestam T. Wound reactions and Aphanomyces astaci growth in crayfish cuticle. Journal of Invertebrate Pathology. 1980;36(2):187–97. http://dx.doi.org/10.1016/0022-2011(80)90023-3.

[32] Schmid-Hempel P, Ebert D. On the evolutionary ecology of specific immune defence. Trends in Ecology & Evolution. 2003;18(1):27–32. http://dx.doi.org/10.1016/S0169-5347(02)00013-7.

[33] Cerenius L, Soderhall K. The prophenoloxidase-activating system in invertebrates. Immunological reviews. 2004;198:116–26. 15199959 10.1111/j.0105-2896.2004.00116.x

[34] Gruber C, Kortet R, Vainikka A, Hyvärinen P, Rantala MJ, Pikkarainen A, et al Variation in Resistance to the Invasive Crayfish Plague and Immune Defence in the Native Noble Crayfish. Annales Zoologici Fennici. 2014;51(4):371–89. doi: 10.5735/086.051.0403

[35] Kusar D, Vrezec A, Ocepek M, Jencic V. Aphanomyces astaci in wild crayfish populations in Slovenia: first report of persistent infection in a stone crayfish Austropotamobius torrentium population. Dis Aquat Organ. 2013;103(2):157–69. doi: 10.3354/dao02567 . 23548366 10.3354/dao02567

[36] Kokko H, Koistinen L, Harlioğlu MM, Makkonen J, Aydın H, Jussila J. Recovering Turkish narrow clawed crayfish (Astacus leptodactylus) populations carryAphanomyces astaci. Knowledge and Management of Aquatic Ecosystems. 2012;(404):12 doi: 10.1051/kmae/2012006

[37] Pedraza-Lara C, Alda F, Carranza S, Doadrio I. Mitochondrial DNA structure of the Iberian populations of the white-clawed crayfish, Austropotamobius italicus italicus (Faxon, 1914). Molecular Phylogenetics and Evolution. 2010;57(1):327–42. http://dx.doi.org/10.1016/j.ympev.2010.06.007. doi: 10.1016/j.ympev.2010.06.007 20601016 10.1016/j.ympev.2010.06.007

